# BEGEV salvage regimen in relapsed/refractory classical Hodgkin lymphoma: a real-life experience

**DOI:** 10.1007/s00432-022-03955-w

**Published:** 2022-03-03

**Authors:** Vittorio Stefoni, Lisa Argnani, Matteo Carella, Beatrice Casadei, Alice Morigi, Ginevra Lolli, Alessandro Broccoli, Cinzia Pellegrini, Laura Nanni, Paolo Elia Coppola, Pier Luigi Zinzani

**Affiliations:** 1 IRCCS-Azienda Ospedaliero-Universitaria di Bologna, Istituto di Ematologia “Seràgnoli”, Bologna, Italy; 2 Dipartimento di Medicina Specialistica, Diagnostica e Sperimentale, Università di Bologna, Bologna, Italy

**Keywords:** BEGEV, Autologous stem cell transplantation, Salvage regimen, Real life, Hodgkin lymphoma

## Abstract

**Purpose:**

One of the most critical issues in the management of Hodgkin lymphoma (HL) patients who resulted as primary relapsed or refractory is to obtain a minimal disease status before autologous stem cell transplantation (ASCT). Finding a salvage regimen able to induce this status without severe toxicity would represent a major achievement in this setting.

**Methods:**

A single‐center retrospective study was conducted to assess effectiveness and safety of BEGEV (bendamustine, gemcitabine, and vinorelbine) regimen as first salvage setting prior to ASCT in HL patients.

**Results:**

Forty-three patients were treated in our institution between October 2017 and November 2020. Median age at BEGEV therapy was 35.0 years (range 17.2– 70.0), and the median time from frontline therapy to the first cycle of BEGEV was 79.5 days (range 4–2267). At the end of treatment, 31 patients achieved a complete response (CR), with an overall response rate of 76.7%. Forty-one patients harvested CD34+ cells and 35/43 (81.4%) patients underwent ASCT. With a median follow‐up of 22 months, 4 CR patients had disease relapse, yielding an estimated disease-free survival of 73.9% at 34 months. The estimated 2‐year progression-free survival was 66.7%. Response to first-line chemotherapy did not significantly influence prognosis.

**Conclusions:**

BEGEV regimen was well tolerated, and reversible haematological toxic effects were the most common adverse events. Real-life data on BEGEV regimen as first salvage setting showed a relevant rate of objective responses and a limited myelotoxicity with no impairment of a subsequent mobilization of peripheral blood stem cells.

## Introduction

After first-line treatment, the standard approach for patients affected by classical Hodgkin lymphoma (cHL) who resulted as refractory to or relapsed consists in the adoption of a salvage chemotherapy which has as target the harvest of autologous stem cells from peripheral blood, followed by high-dose chemotherapy and, if feasible, autologous stem cell transplantation (ASCT). This sequence results in a long-term progression-free survival (PFS) for 50–60% of subjects with a chemosensitive relapse (Linch et al. 1993; Schmitz et al. 2002), but this therapeutic approach does not induce same results for patients with primary chemorefractory disease, yielding a long-term survival which rarely exceeds 15–17% (Sureda et al. 2005; Arai et al. 2013). Disease recurrence still remains the principal cause of ASCT failure, and a disease progression within 6 months from high-dose conditioning results as a critical negative prognostic factor for patients’ outcome (Sureda et al. 2005).

Given these premises, the goal for physicians and researchers is to improve outcomes following high-dose regimens and ASCT, and to provide an actual chance of cure for relapsed/refractory patients (Broccoli and Zinzani 2019). In particular, one of the most critical issues in the management of HL patients who resulted as primary relapsed or refractory is to obtain a minimal disease status, i.e., achieving a positron emission tomography (PET)-negative status before undergoing ASCT. Finding a salvage regimen able to induce this status without severe toxicity would represent a major conquest in this setting (Moskowitz et al. 2010a, b; Devillier et al. 2012; Gentzler et al. 2014).

The treatment of choice for cHL patients who resulted as primary refractory or relapsed is platinum‐based and ifosfamide‐containing regimens even if the complete remission rate (required for ASCT) is lower than 30–35% (Santoro et al. 2007). To note, the scheduling of an adequate supportive treatment is strongly recommended.

In 2016, a multicenter phase 2 trial on the combination of bendamustine, gemcitabine, and vinorelbine (BEGEV regimen) was reported with promising results in 59 patients with relapsed or refractory HL (Santoro et al. 2016). The final overall response rate (ORR) was 83%. In details, after 4 cycles of treatment, 43 patients (73%) achieved a complete response (CR) and 6 patients (10%) a partial response (PR). Grade 3–4 thrombocytopenia and neutropenia were the most frequent haematological adverse events, occurring in 13.5% of patients each, allowing unfailing regimen administration. To note, peripheral blood stem cell (PBSC) mobilization and harvest were performed in 96% of patients: 88% of these patients underwent ASCT (Santoro et al. 2016). The regimen induced in the total study population a 2-year estimated PFS rate of 62.2% and an overall survival (OS) rate of 77.6%, respectively, with a median follow-up of 29 months (Santoro et al. 2016). Trial results provided a strong rationale for further use of the BEGEV regimen and we adopted it in our institution. The aim of the present report was to analyse and evaluate our clinical experience with BEGEV regimen as salvage regimen prior to ASCT in primary relapsed/refractory cHL patients in a real-life setting.

## Patients and methods

A single‐center observational retrospective study was conducted. Consecutive patients with cHL who were refractory to or had relapse after receiving one previous chemotherapy line and subsequently received BEGEV regimen scheduled as salvage regimen prior to ASCT were eligible.

The local Ethical Committee approved this observational study along with our institutional board (CE AVEC di Bologna, ID 428/2021/Oss AOUBo deliberation of 2 July 2021). All patients signed the informed consent and we enrolled them consecutively to avoid selection bias. As for the retrospective design of the study, we received an authorization to analyse data also of patients who resulted as deceased or lost to follow-up at the time of data collection. The study was conducted in respect of the Declaration of Helsinki and its later amendments.

The BEGEV regimen administered was as follows: gemcitabine 800 mg/m^2^ and prednisolone 100 mg per day on days 1 and 4, vinorelbine 20 mg/m^2^ on day 1, and bendamustine 90 mg/m^2^ on days 2 and 3. Effectiveness was assessed as ORR (sum of CR and PR rates), PFS, disease-free survival (DFS) and OS. OS for all patients was calculated from start of BEGEV to the last follow-up or death for any cause; PFS for all patients was calculated from start of BEGEV to the first disease progression or death; DFS was determined in all CR patients as the time between the first documented response and the first disease relapse, or death as a result of lymphoma or acute treatment toxicity. Staging and restaging assessments with imaging were performed before, after 2 cycles, after 4 cycles, before and after ASCT; following completion of the treatment, PET and computed tomography scans were performed every 6 months for the first 2 years and every 12 months for further 3 years. Responses were classified according to the International Workshop for Response Criteria for non‐Hodgkin lymphomas. Safety and tolerability were assessed by recording type, incidence, and severity of any adverse events (AEs) (assessed with National Cancer Institute Common Terminology Criteria of AEs v4.0). No formal sample size estimation and power calculation were made for this observational retrospective study as we consecutively enrolled all cHL who were refractory to or had relapse after receiving one previous chemotherapy line and subsequently underwent BEGEV regimen with salvage intent. Patients’ characteristics and demographics were analysed with descriptive statistics, time-to-event functions were estimated with the Kaplan–Meier method and comparisons were made with the log-rank test. *p* value for significativity was set at 0.05. All analyses were conducted with Stata 11 (StataCorp LP, TX).

## Results

In total, 43 patients (25 men and 18 women) were treated in our institution between October 2017 and November 2020. Patient characteristics are shown in Table 1. Briefly, median age at BEGEV was 35.0 years (range 17.2–70.0 years), and the median time from frontline therapy to first cycle of BEGEV was 79.5 days (range 4–2267 days). Forty patients (93.0%) had received ABVD (doxorubicin, bleomycin vinblastine, and dacarbazine) as frontline therapy, while one patient had received RCOMP (prednisone, cyclophosphamide, vincristine, doxorubicin, and rituximab), one COPP-ABV (cyclophosphamide, vincristine, procarbazine, prednisone, doxorubicin, bleomycin, and vinblastine), and one VBM (vinblastine, bleomycin, and methotrexate). Twenty-three patients were primary refractory. Right before BEGEV, all patients had an ECOG performance status score of 0, and disease stage was II in 28 subjects, III in 6 patients, and IV in 9 ones (with a predominance of pulmonary involvement), respectively.

**Table 1:** Patients’ characteristics

	Total population
Patients, *n*	43
Males, *n* (%) Females, *n* (%)	25 (58.1) 18 (41.9)
Median age at diagnosis, years (range)	32.9 (13.8–69.3)
Stage at diagnosis, *n* (%)	
II III IV	16 (37.2) 8 (18.6) 9 (20.9)
Extranodal site at diagnosis, *n* (%)	
Total Lung Lung and bone Liver and bone Bone Bone marrow	19 (44.2) 7 (16.3) 4 (9.3) 4 (9.3) 2 (4.7) 2 (4.7)
First line, *n* (%)	
ABVD Other	40 (93.0) 3 (7.0)
Outcome of first line, *n* (%)	
Refractory Relapsed	23 (53.5) 20 (46.5)
Stage at BEGEV, *n* (%)	
II III IV	28 (65.1) 6 (13.9) 9 (20.9)
ECOG PS, *n* (%)	
0 1 2	43 (100) – –
Median age at BEGEV, years (range)	35.0 (17.2–70.0)
Response to BEGEV, *n* (%)	
CR PR SD PD	31 (72.1) 2 (4.7) 2 (4.7) 8 (18.6)

*ABVD* doxorubicin, bleomycin vinblastine, and dacarbazine, *BEGEV* bendamustine, gemcitabine, and vinorelbine, *CR* complete response, *ECOG PS* Eastern Cooperative Oncology Group performance status, *SD* stable disease, *RCOMP* doxorubicin, cyclophosphamide, vincristine, and prednisone plus rituximab

Patients received 2–4 cycles (median 3 cycles) of the BEGEV regimen administered every 21 days in the outpatient clinic. Patients have been received 2 or 3 cycles instead of 4 in two different situations: (1) patients who rapidly progressed and thus stopped the treatment with BEGEV; (2) patients in complete metabolic response at the interim restaging but with signs of PS worsening and thus were rapidly addressed to ASCT. Growth‐stimulating factors were administered at each cycle to prevent neutropenia. In addition, patients received pneumocystis pneumonia prophylaxis and antiemetics (all according to our institutional guidelines). Treatment was interrupted in case of disease progression or unacceptable toxicity. After BEGEV regimen patients received consolidation with ASCT utilizing BEAM regimen (BCNU, etoposide, cytarabine, and melphalan) followed by reinfusion of at least 2 × 10^6^ per kilogram of CD34+ cells. Collection of CD34+ cells was performed usually after the second cycle (range 1–3); the median total yield of CD34+ cells per kilogram was 7.26 × 10^6^ (range 1.92–25.00 × 10^6^ cells).

At the end of BEGEV, 31 (72.1%) out of the 43 patients achieved a best response of CR, 2 PR, 2 a stable disease (SD) and the remaining two patients showed progression of disease with an ORR of 76.7%.

All the patients were scheduled ASCT. Forty-one out of the 43 patients harvested CD34+ cells and 35/43 (81.4%) patients underwent ASCT. At the PET evaluation post-ASCT all patients showed a CR including also the two patients who underwent ASCT with a best response of SD (they achieved a CR subsequently to transplant).

**Fig. 1:**
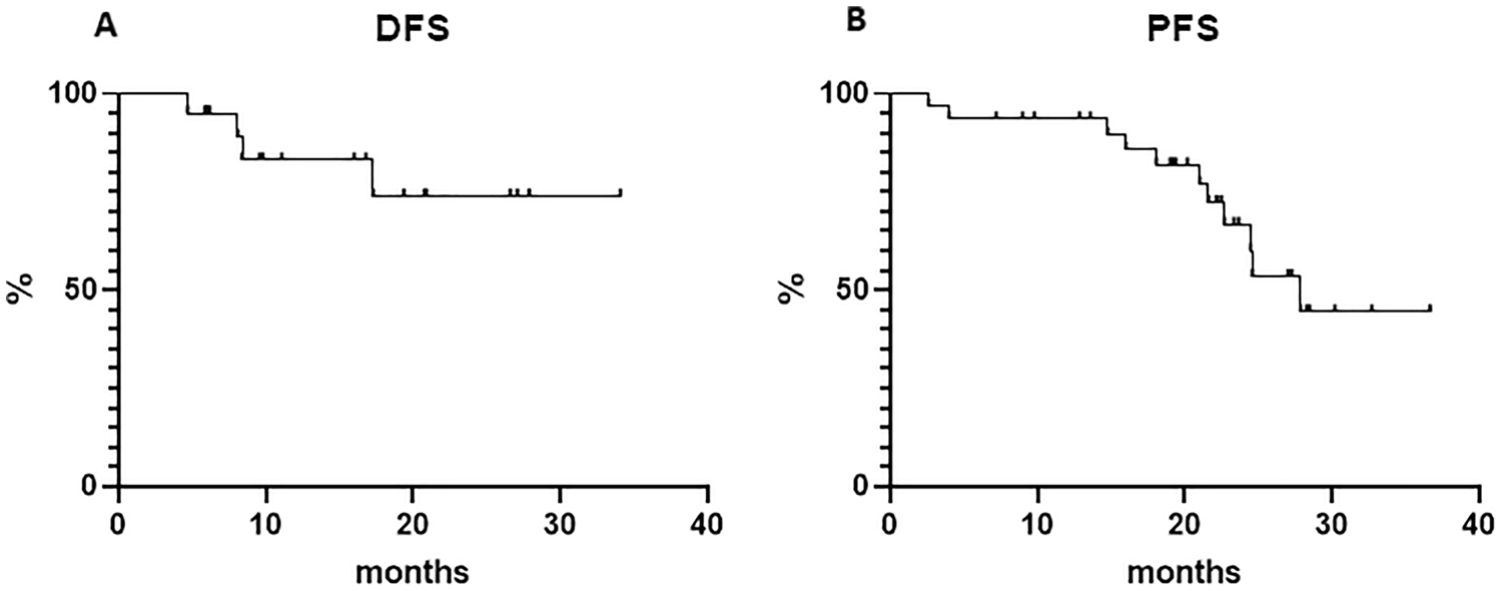
Disease-free survival (**a**) and progression-free survival (**b**)

With a median follow‐up time of 22 months (range 6–26), 4 (after 5, 8, 8, and 17 months, respectively) of 31 (12.9%) CR patients had disease relapse, yielding an estimated DFS of 73.9% at 34 months (median not reached, Fig. 1a). The estimated OS for the whole cohort was 100% at 36 months. The estimated 3-year for the whole cohort PFS was 44.4% (median reached at 27.9 months, Fig. 1b).

Patient’s status in respect to the first-line treatment, i.e., refractory versus relapsed, did not affect outcomes (*p* > 0.1). Twelve patients received subsequent therapy, only in case of progression or relapse. In detail, 11 patients received brentuximab vedotin and one patient underwent bendamustine. No responder patients received consolidation or maintenance therapy.

The BEGEV regimen was well tolerated by most patients, and reversible haematological toxic effects were the most common AEs.

Grade ≥ 3 AEs were reported in 16 (37.2%) patients, and the most common AEs were neutropenia (14, 32.6%), thrombocytopenia (13, 30.2%), and febrile neutropenia (2, 4.7%). Another AE of clinical interest was grade 3 pneumonia/pneumonitis in 1 (2.3%) patient. No secondary haematological malignancies were observed. All the other extra-haematological toxicities were grade ≤ 2 and easily manageable: most frequent ones were grade 1/2 asthenia (7.0%), grade 1 diarrhoea (6.5%), and grade 1 nausea (5.0%). No treatment‐related deaths occurred.

## Discussion

Ideally, a first salvage regimen should present some peculiar characteristics. First of all, it has to induce an effective disease control, which means to have the opportunity of achieving high CR rates. No less important, the choice of the salvage must fall on the one that allows an adequate mobilization of PBSC without resorting to additional chemotherapy. In third instance, the adopted regimen has to show also an acceptable safety profile, i.e., without or limited myelotoxic events, avoiding a harmful peripheral cytopenia.

The current chemotherapy regimens in the first salvage setting usually lead to a significant myelosuppression, risk of infection, and gastrointestinal toxicity in patients. In addition, rate of CR induced by these regimens ranges from 20 to 60% (Moskowitz et al. 2001; Josting et al. 2002; Baetz et al. 2003; Santoro et al. 2007). We showed that BEGEV regimen induced a relevant rate of objective responses in patients with primary relapsed or refractory cHL (consisting in an encouraging rate of metabolic CR) as a proof of its effectiveness.

The BEGEV regimen is also safe in comparison with other conventional chemotherapeutic salvage regimens which reported grade 3–4 neutropenia and thrombocytopenia in 60–90% of cases versus 37% and 30% in our report, respectively (Santoro et al. 2007), and in respect of the results of the phase 3 trial which compared additional sequential high-dose chemotherapy with conventional one (Josting et al. 2010). In fact, limited myelotoxicity occurred in our study population and the subsequent mobilization of PBSC was not impaired.

We registered an ORR of 76.7%, and patients harvested an adequate amount of CD34+ cells. BEGEV showed activity both in relapsed patients and in patients with primary refractory disease. Similar results have been obtained in the phase 2 study for the same treatment context and in a population with overlapping clinical characteristics (Santoro et al. 2016). Recently, Santoro and colleagues reported the long-term efficacy data showing a long duration of response without any late toxicity within a 5-year period (Santoro et al. 2020).

Our study has, however, some limitations as its monocentric and retrospective characteristics and the fact that our rationale is based on the results of a phase 2 study with a limited sample size and not on results of a phase 3 trial.

## Conclusion

Many novel agents are and will be available in the near future even in the pre-transplantation therapy setting of cHL: as direct comparisons are currently not feasible, our results support the adoption of the BEGEV regimen in the real-life clinical practice.

As future research directions, combinations of these novel agents with BEGEV and/or other regimens would be decisive to establish the best salvage pathway in the setting of primary relapsed or refractory cHL.

Our report represents the first confirmation in a real-world context on the effective and safe role of BEGEV in the setting of early relapsed/primary refractory cHL patients. BEGEV as effective salvage regimen induced a CR in a high proportion of patients with no impairment of a subsequent mobilization of peripheral blood stem cells.

## Abbreviations

AE: Adverse event
ASCT: Autologous stem cell transplantation
BEAM: BCNU, etoposide, cytarabine, and melphalan
BEGEV: Bendamustine, gemcitabine, and vinorelbine
cHL: Classical Hodgkin lymphoma
CR: Complete response
DFS: Disease-free survival
ORR: Overall response rate
OS: Overall survival
PBSC: Peripheral blood stem cell
PET: Positron emission tomography
PFS: Progression-free survival
PR: Partial response
SD: Stable disease
